# Validating Recipients of Pediatric Solid Organ Transplant Using Administrative Healthcare Data

**DOI:** 10.1111/petr.70245

**Published:** 2025-12-08

**Authors:** Simran Aggarwal, Kyla Naylor, Rulan S. Parekh, Jovanka Vasilevska‐Ristovska, Stephanie N. Dixon, Yuguang Kang, Rahul Chanchlani

**Affiliations:** ^1^ Division of Nephrology, Department of Pediatrics, McMaster Children's Hospital McMaster University Hamilton Ontario Canada; ^2^ ICES Toronto Ontario Canada; ^3^ London Health Sciences Centre Research Institute London Ontario Canada; ^4^ Department of Medicine and Epidemiology and Biostatistics Western University London Ontario Canada; ^5^ Women's College Hospital Toronto Ontario Canada; ^6^ Hospital for Sick Children Toronto Ontario Canada; ^7^ University of Toronto Toronto Ontario Canada; ^8^ Department of Epidemiology and Biostatistics Western University London Ontario Canada

**Keywords:** health administrative database, pediatric transplant, solid organ transplant, validation study

## Abstract

**Background:**

Health administrative datasets have the potential to provide valuable insights into pediatric solid organ transplantation; however, validation is necessary to ensure their accuracy. This study aimed to assess the validity of administrative data by comparing it to direct transplant records from a major pediatric transplant center in Ontario, Canada (1991–2011).

**Methods:**

Using linked administrative healthcare databases, we conducted a retrospective analysis to evaluate the validity of physician billing claims and hospital diagnostic and procedural codes in identifying pediatric solid organ transplants. Sensitivity and positive predictive value (PPV) were calculated for various algorithms.

**Results:**

During the study period, a total of 347 kidney, 250 liver, 200 heart, and 28 lung transplants were performed. The best algorithm for identifying these transplants utilized hospital procedural codes from the Canadian Institute for Health Information Discharge Abstract Database. Compared to transplant center records, these codes demonstrated a sensitivity of 91% (95% CI: 89–93) and PPV of 93% (95% CI: 91–95) when including all organ types, and performed similarly well when evaluating individual organ types.

**Conclusion:**

This study is the first to validate administrative data for identifying pediatric solid organ transplant recipients, demonstrating the reliability of procedural codes for population‐level health research in this domain.

## Graphical abstract

This study validated the use of health administrative databases to identify pediatric solid organ transplant recipients. The results indicate that procedural codes offer a reliable method for patient identification, demonstrating their reliability in conducting population‐level health research in this domain.

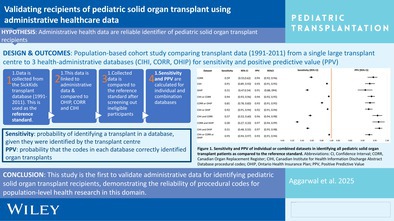

**Abbreviations**
CI: confidence interval
CIHI: Canadian Institute for Health Information
CIHI‐DAD: Canadian Institute for Health Information—Discharge Abstract Database
CIHI‐DAD‐p: Canadian Institute for Health Information—Discharge Abstract Database procedural codes
CORR: Canadian Organ Replacement Registry
OHIP: Ontario Health Insurance Plan
PPV: positive predictive value
SAS: Statistical Analysis Software

## 1 Introduction

Pediatric organ transplantation remains the sole curative therapeutic procedure for most end‐stage diseases with organ failure [1, 2, 3]. In recent years, the field of pediatric solid organ transplantation has seen remarkable advancements, with significant improvements in survival rates and life expectancy [1, 2, 3, 4, 5]. As an increasing number of children with transplants reach adulthood, there is growing interest among clinicians, policymakers, and families, in understanding their long‐term health outcomes. However, research in this area faces significant challenges, including small cohort sizes, loss to follow‐up, and insufficient long‐term data [1, 4]. Additionally, studies are often constrained by their reliance on data from individual transplant centers, which may have varying practices and limitations in data collection [6].

Population‐based studies using administrative healthcare data can address many challenges. Originally developed for administrative and billing purposes, these data, generated from patient encounters, can be utilized to study healthcare delivery, outcomes, and costs [7]. Health administrative databases capture variables not routinely collected by transplant centers, enabling the evaluation of otherwise inaccessible outcomes [6]. These databases also support long‐term follow‐up, minimize follow‐up loss, and provide large sample sizes, increasing statistical power, and generalizability of results [2, 8].

However, when using these data sources for clinical or health services research, the validity of the research hinges on the accuracy of the recorded diagnostic and procedural codes [9]. Validation of the accuracy of these administrative codes is crucial to ensure that research and healthcare decisions are informed by reliable data [10, 11]. Validated algorithms to identify pediatric solid organ transplant recipients within health administrative data are currently lacking. Therefore, we conducted a study to evaluate the validity of using various codes available in health administrative databases for identifying pediatric solid organ transplant recipients compared to information collected directly from medical charts from a pediatric transplant center.

## 2 Methods

### 2.1 Study Design and Setting

We conducted a population‐based cohort study using administrative healthcare databases. These datasets were linked using unique encoded identifiers and analyzed at ICES in Ontario, Canada (ices.on.ca). ICES is an independent, non‐profit research institute whose legal status under Ontario's health information privacy law allows it to collect and analyze healthcare and demographic data, without consent, for health system evaluation and improvement. The use of all other data in this project was authorized under section 45 of Ontario's Personal Health Information Protection Act, which does not require review by a Research Ethics Board. The use of the pediatric transplant data was approved by the Research Ethics Board at the Hospital for Sick Children (REB # 1000034626). Our study is reported in accordance with the Reporting of Studies Conducted Using Observational Routinely Collected Health Data (RECORD) guidelines (Appendix S1) [12].

### 2.2 Data Sources

We used data from the largest pediatric transplant center in Ontario, Canada (Hospital for Sick Children, Toronto [SickKids]), which provided information on pediatric kidney, lung, liver and heart transplants from July 1, 1991 to December 31, 2011. The Hospital for Sick Children performs nearly all pediatric solid organ transplants in Ontario, with the exception of kidney transplants in adolescents, which were also performed at London Health Sciences Centre during this time period. This transplant center also ranks among the largest on the continent, with case volumes reported to be within the top 5% of centers in North America [13, 14].

Transplant data from SickKids, including patient‐level information (such as OHIP numbers and other identifiers), was collected by trained abstractors from the transplant center's electronic registry and the hospital clinical electronic health record. These identifiers were then used to link patients to provincial health administrative databases, enabling comparison between the number of transplants reported by the hospital and those captured in administrative data sources. We considered information from the transplant center to be the reference standard and compared it to information derived from three different healthcare administrative databases; these included a provincial physician billing database (i.e., Ontario Health Insurance Plan [OHIP]), and national databases (i.e., Canadian Organ Replacement Register [CORR], Canadian Institute for Health Information—Distract Abstraction Database [CIHI‐DAD] which provided procedural [CIHI‐DAD‐p] and diagnostic codes [CIHI‐DAD‐d]). The OHIP database contains records of physician billings for outpatient and inpatient services. The CORR is a pan‐Canadian database that collects information on all Canadians under transplant programs, organ donation organizations and independent health facilities to track patients from transplantation to death [15]. CIHI‐DAD captures information on diagnoses and procedures occurring during a hospital admission; data is received directly from all acute care facilities and reporting is mandatory in Ontario. CIHI‐DAD data quality is assessed on a continuous basis and is > 99% complete [16]. Appendix S2 in Supporting Information includes descriptions of each database and administrative definitions used.

### 2.3 Population

Based on data availability, we included pediatric solid organ transplant recipients from January 1, 1991 to December 31, 2011. We defined a solid organ transplant as a kidney‐only, lung‐only, liver‐only or heart‐only transplant. Patients undergoing multi‐organ, liver‐bowel, or intestinal transplants were excluded because each group contained fewer than 6 patients, and ICES policy prohibits reporting cell sizes < 6 to protect patient confidentiality. If an individual had multiple transplants, we restricted it to the first record of any solid organ transplant. After data cleaning (missing or invalid ICES key number [unique patient identifier created by ICES] [17], age or sex, and death on or before the cohort entry date), we excluded solid organ transplant recipients who were not residents of Ontario, Canada, aged 18 years or older on the date of transplant, received simultaneous multi‐organ transplants, or had transplant surgery not performed at SickKids (13% to 28.6%) (Figure S1). Patients who were not residents of Ontario were excluded because Ontario's administrative healthcare data can only reliably capture individuals with a valid Ontario healthcare number.

### 2.4 Statistical Methods

#### 2.4.1 Primary Analysis

We compared data from each of the administrative databases to the number of organ transplants reported by the transplant center during the study period. To count as a concordant transplant between the reference standard and the Ontario‐wide administrative databases (e.g., CORR), we ensured the ICES key numbers (i.e., unique patient identifier) aligned, and the transplant dates were within 7 days of each other.

We calculated the sensitivity (95% CI), defining it as the probability of identifying a transplant in the administrative data, given that they were identified by the transplant center. In addition, we calculated the positive predictive value (PPV, 95% CI), defining it as the probability that the codes in each database correctly identified organ transplants.

We also calculated sensitivity and PPV using various combinations of the above databases, for a total of 7 combinations, including: “CIHI‐DAD‐p or CORR”, “CORR or OHIP”, “CIHI‐DAD‐p or OHIP”, “CIHI‐DAD‐p and CORR”, “CIHI‐DAD‐p and OHIP”, “CORR and OHIP”, and “CIH‐DADI‐p or CORR or OHIP” (Figure S2).

#### 2.4.2 Additional Analysis

We conducted an additional analysis to capture the sensitivity and PPV for the three individual datasets for solid organ transplants stratified by individuals who entered the cohort before and after April 1, 2002. This sought to analyze any changes in accuracy in relation to the switch from the International Classification of Disease 9th revision (ICD‐9) to ICD‐10 codes, and the switch from Canadian Classification of Diagnostic, Therapeutic, and Surgical Procedures (CCP) to the Canadian Classification of Health Interventions (CCI) [18, 19]. Kidney transplants, being the largest subgroup, were further selected for the same analysis.

We also analyzed the concordance between transplant dates across CIHI‐DAD‐p, OHIP and CORR, as compared to the dates reported by the transplant center. The mean (standard deviation) and median (25th, 75th percentile) were calculated as measures of central tendency to assess for similarity between transplant dates reported by each database versus the transplant center.

All analyses were conducted using SAS version 9.4 (SAS Institute, Cary, NC).

## 3 Results

During the study period, there were a total of 825 solid organ transplants reported by the transplant center, including 347 kidney (42%), 250 liver (30%), 200 heart (24%), and 28 lung transplants (3%). In comparison to the reference standard, CORR identified 514 transplants, CIHI‐DAD‐p identified 804 transplants, CIHI‐DAD‐d identified 654 transplants and OHIP identified 459 transplants (Figure S1). See Table S1 for contingency tables.

### 3.1 All Solid Organ Transplants

**FIGURE 1:**
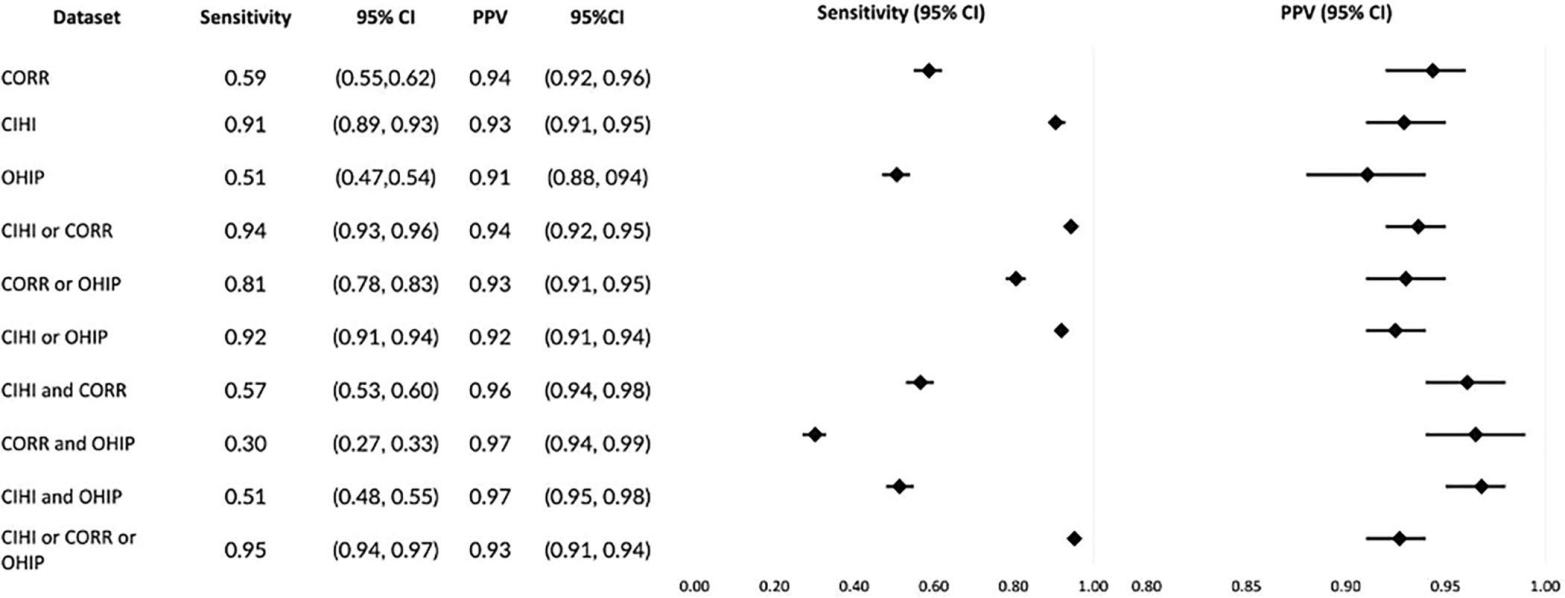
Sensitivity and PPV of individual or combined datasets in identifying all pediatric solid organ transplant patients as compared to the reference standard. CI, Confidence Interval; CORR, Canadian Organ Replacement Register; CIHI, Canadian Institute for Health Information Discharge Abstract Database procedural codes; OHIP, Ontario Health Insurance Plan; PPV, Positive Predictive Value.

The probability of identifying recipients of all solid organ transplants in CORR, CIHI‐DAD‐p, and OHIP, given they were identified by the transplant center (sensitivity), was 59%, 91%, and 51% respectively. The probability that the database code correctly identified a transplant recipient (PPV) in CORR, CIHI‐DAD‐p, and OHIP was 94%, 93%, and 91% respectively. CIHI‐DAD diagnostic codes were removed from analysis as < 6 individuals with a CIHI‐DAD diagnosis code aligned with the transplant date in the reference standard. Across all 10 algorithms, sensitivity ranged from 30% to 95%, with “CORR and OHIP” lowest and “CIHI‐DAD‐p or CORR or OHIP” the highest. PPV ranged from 91% to 97%, with “OHIP” lowest and both “CORR and OHIP” and “CIHI‐DAD‐p and OHIP” highest. CIHI‐DAD‐p alone was only slightly lower in both sensitivity and PPV as compared to the highest ranking algorithms (Figure 1).

**FIGURE 2:**
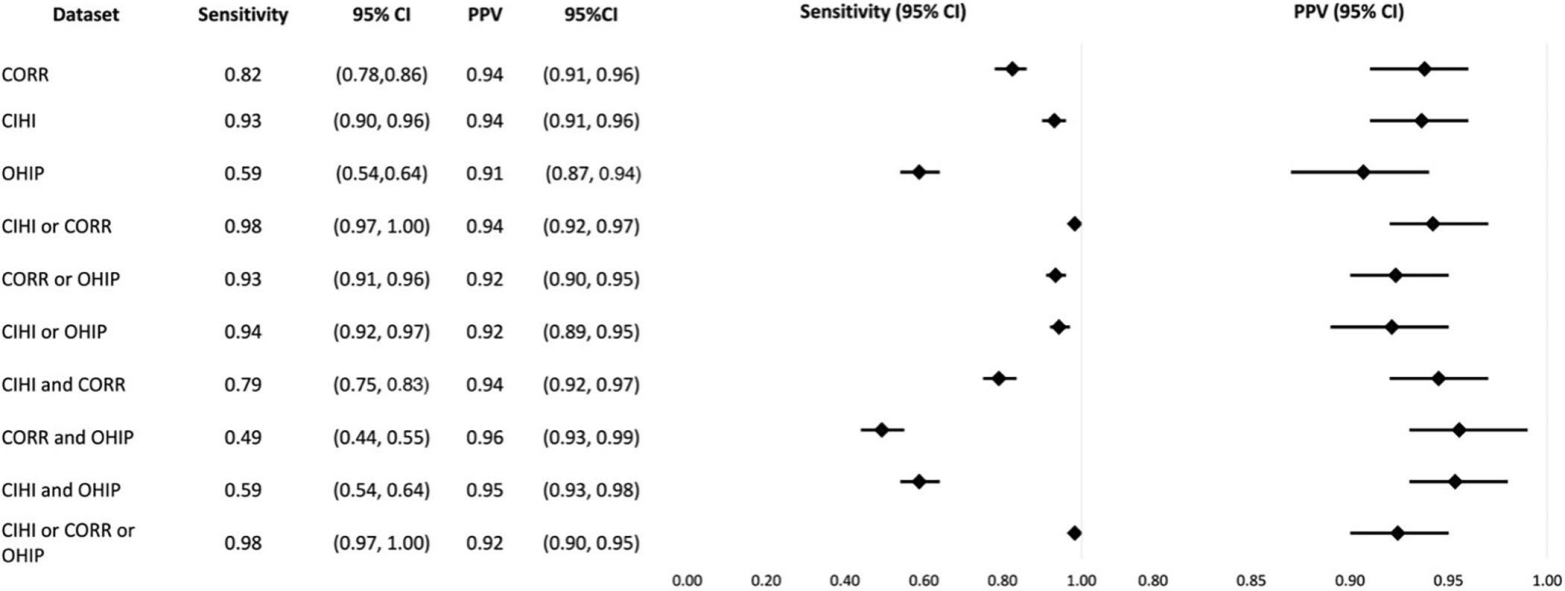
Sensitivity and PPV of individual or combined datasets in identifying all pediatric kidney transplant patients as compared to the reference standard. CI, Confidence Interval; CORR, Canadian Organ Replacement Register; CIHI, Canadian Institute for Health Information Discharge Abstract Database procedural codes; OHIP, Ontario Health Insurance Plan; PPV, Positive Predictive Value.

**FIGURE 3:**
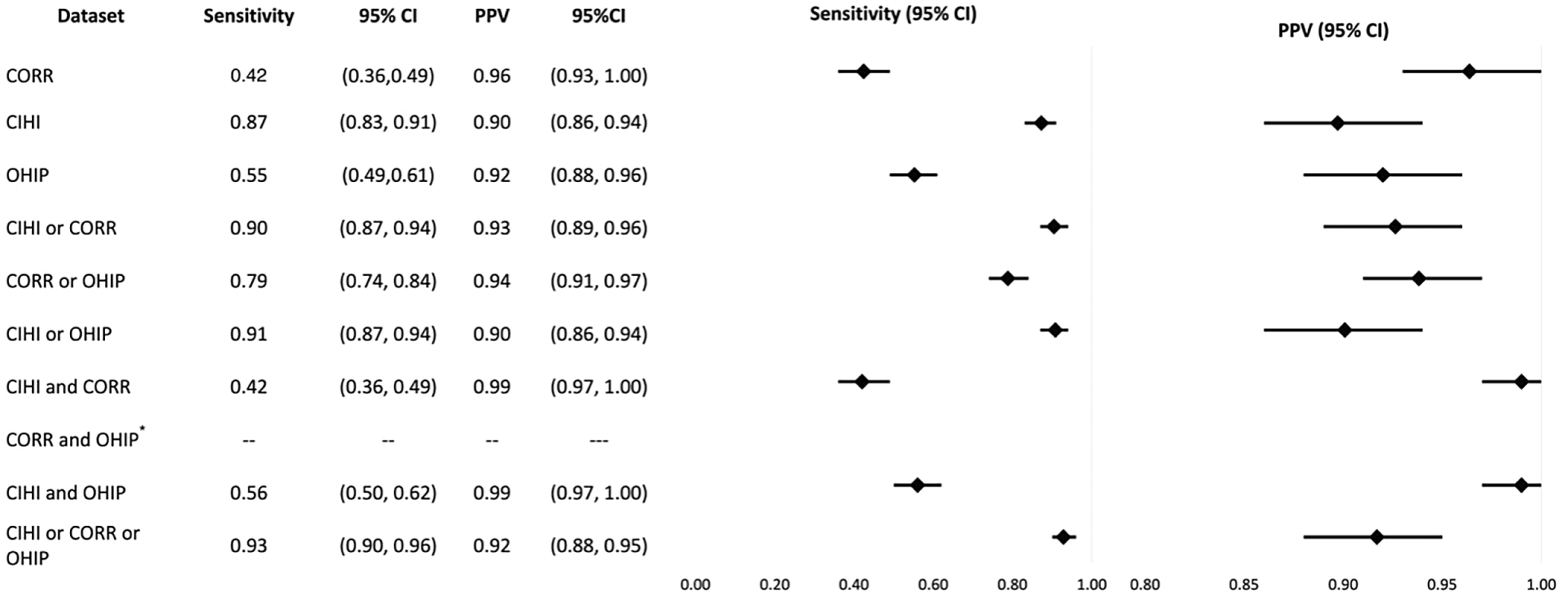
Sensitivity and PPV of individual or combined datasets in identifying all pediatric liver transplant patients as compared to the reference standard. *Due to small cell counts, the sensitivity and positive predictive value could not be calculated for the CORR and OHIP algorithm. CI, Confidence Interval; CORR, Canadian Organ Replacement Register; CIHI, Canadian Institute for Health Information Discharge Abstract Database procedural codes; OHIP, Ontario Health Insurance Plan; PPV, Positive Predictive Value.

**FIGURE 4:**
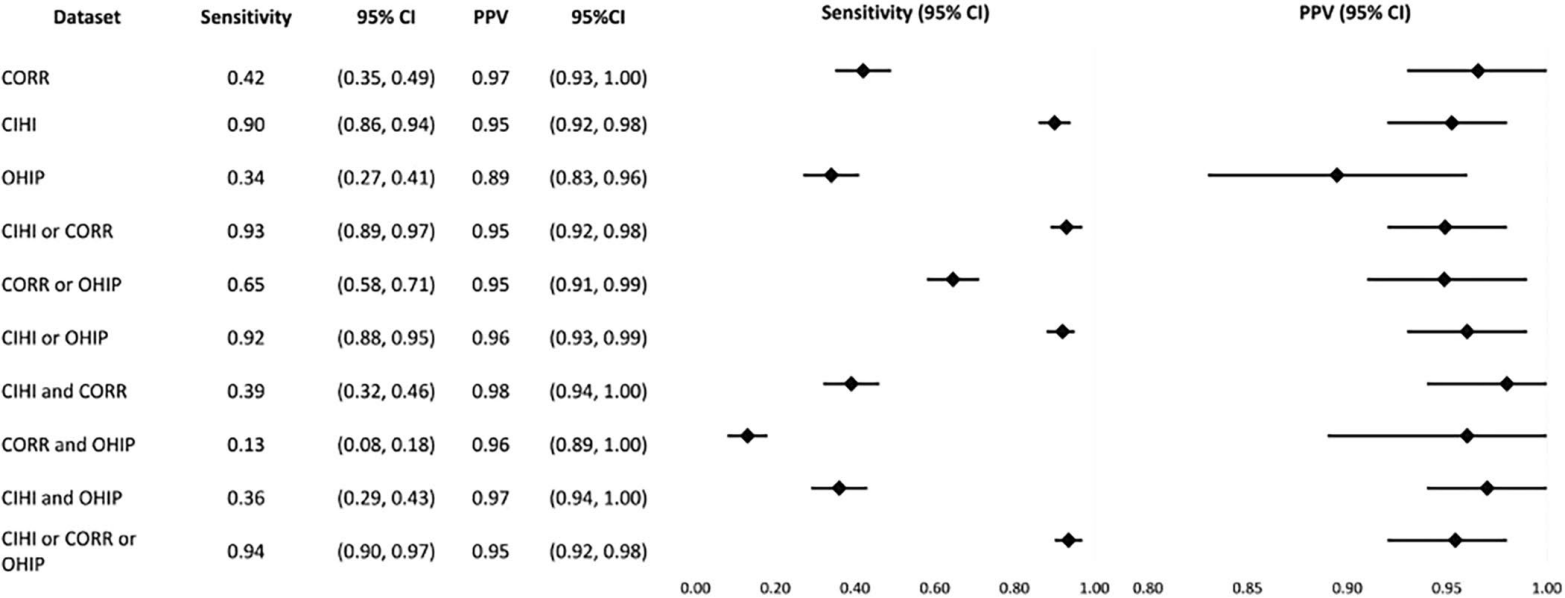
Sensitivity and PPV of individual or combined datasets in identifying all pediatric heart transplant patients as compared to the reference standard. CI, Confidence Interval; CORR, Canadian Organ Replacement Register; CIHI, Canadian Institute for Health Information Discharge Abstract Database procedural codes; OHIP, Ontario Health Insurance Plan; PPV, Positive Predictive Value.

**FIGURE 5:**
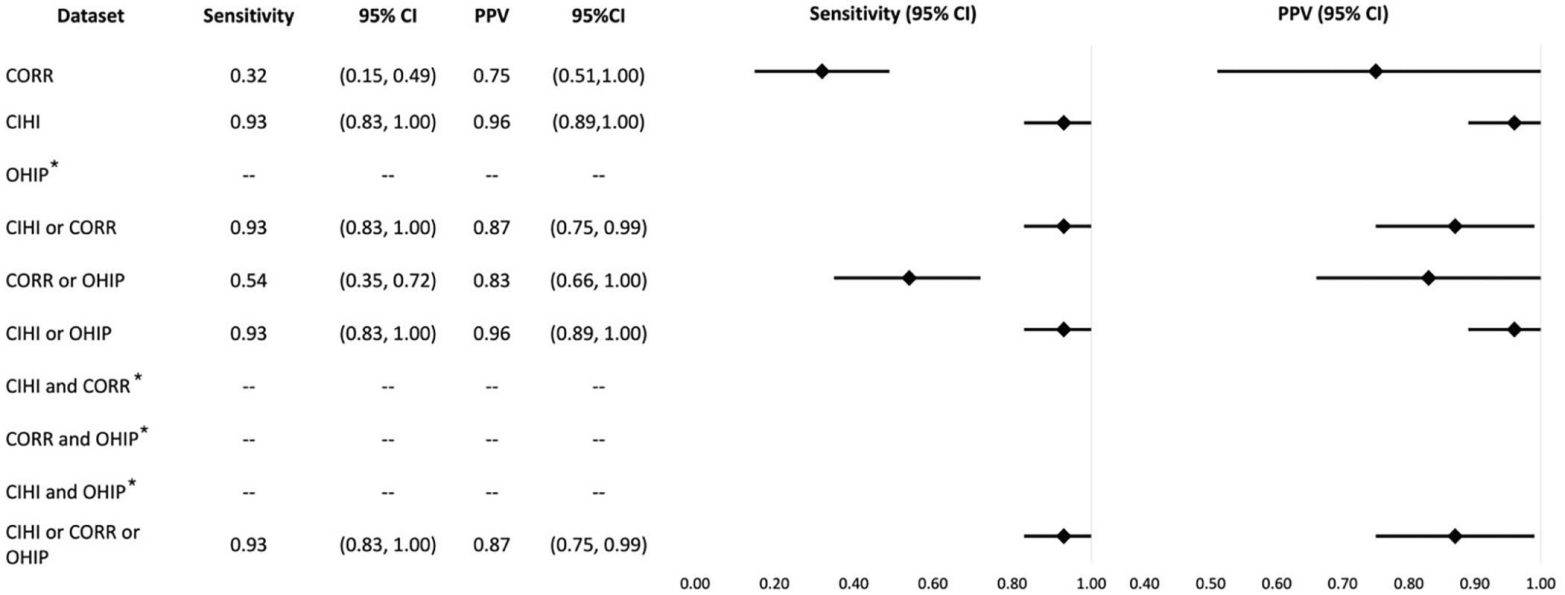
Sensitivity and PPV of individual or combined datasets in identifying pediatric lung transplant patients as compared to the reference standard. *Due to small cell counts, the sensitivity and positive predictive value could not be calculated. Abbreviations: CI, Confidence Interval; CORR, Canadian Organ Replacement Register; CIHI, Canadian Institute for Health Information Discharge Abstract Database procedural codes; OHIP, Ontario Health Insurance Plan; PPV, Positive Predictive Value.

While sensitivities varied largely between algorithms and types of transplants studied, sensitivity for OHIP alone was overall poor, ranging between 34‐59%. CORR also displayed poor sensitivity for all algorithms except kidney (which had a sensitivity of 82%), ranging between 32% and 59% (Figures 1, 2, 3, 4, 5).

### 3.2 Kidney Transplants

The sensitivity of CORR, CIHI‐DAD‐p, and OHIP was 82%, 93%, and 59% respectively, while the PPV was 94%, 94%, and 91%. Across all 10 algorithms, sensitivity ranged from 49% to 98%, with “CORR and OHIP” lowest and “CIHI‐DAD‐p or CORR or OHIP” and “CIHI‐DAD‐p or CORR” highest. PPV ranged from 91% to 96%, with “OHIP” lowest and “CORR and OHIP” highest. Similar to all solid organ transplants, CIHI‐DAD‐p alone had sensitivity and PPV comparable to the top‐performing algorithms, followed closely by CORR (Figure 2).

### 3.3 Liver Transplants

The sensitivity of CORR, CIHI‐DAD‐p, and OHIP was 42%, 87%, and 55% respectively, while the PPV was 96%, 90%, and 92%. Across all 10 algorithms, sensitivity ranged from 42% to 93%, with “CORR” and “CIHI‐DAD‐p and CORR” having the lowest and “CIHI‐DAD‐p or CORR or OHIP” having the highest. PPV ranged from 90% to 99%, with “CIHI‐DAD‐p” and “CIHI‐DAD‐p or OHIP” having the lowest and “CIHI‐DAD‐p and CORR” and “CIHI‐DAD‐p and OHIP” having the highest. Again, CIHI‐DAD‐p alone had similar sensitivity and PPV compared to “CIHI‐DAD‐p or CORR or OHIP” (Figure 3).

### 3.4 Heart Transplants

The sensitivity of CORR, CIHI‐DAD‐p, and OHIP was 42%, 90%, and 34% respectively, while the PPV was 97%, 95%, and 89%. Across all 10 algorithms, sensitivity ranged from 13% to 94%, with “CORR and OHIP” lowest and “CIHI‐DAD‐p or CORR or OHIP” highest. PPV ranged from 89% to 98%, with “OHIP” lowest and “CIHI‐DAD‐p and CORR” highest. CIHI‐DAD‐p alone had comparable sensitivity and PPV to the best performing algorithms (Figure 4).

### 3.5 Lung Transplants

The sensitivity of CORR and CIHI‐DAD‐p was 32%, 93% respectively, while the PPV was 75% and 96%. Sensitivity and PPV for OHIP could not be reported given the low number of identified recipients. Across all 10 algorithms, sensitivity ranged from 32% to 93%, with “CORR” lowest and “CIHI‐DAD‐p”, “CIHI‐DAD‐p or CORR”, “CIHI‐DAD‐p or OHIP”, and “CIHI‐DAD‐p or CORR or OHIP” highest. PPV ranged from 75% to 96%, with “CORR” lowest and “CIHI‐DAD‐p” and “CIHI‐DAD‐p or OHIP” highest. Overall, CIHI‐DAD‐p had the highest sensitivity, whether alone or in combination with OHIP or CORR. Within these algorithms, “CIHI‐DAD‐p” and “CIHI‐DAD‐p or OHIP” had the highest PPV (Figure 5).

### 3.6 Transplant Date Concordance

Date concordance analysis revealed relatively small discrepancies among transplant dates reported in CIHI‐DAD‐p, OHIP and CORR as compared to the transplant center. The average discrepancies were 0.03 days (SD 0.48) for CORR, 0.03 days (SD 0.68) for CIHI‐DAD‐p and 0.03 days (SD 0.69) for OHIP. The mean absolute differences were 0.14 days (SD 0.46) for CORR, 0.18 days (SD 0.66) for CIHI‐DAD‐p, and 0.20 days (SD 0.66) for OHIP, with an IQR of 0 to 0. The median absolute difference between the recorded transplant date at the transplant center was 0 days (IQR, 0 to 0) for all algorithms.

### 3.7 Additional Analysis

Both CIHI‐DAD‐p and OHIP demonstrated higher sensitivity and PPV in cohorts that entered the study after April 1 2002, as compared to before. In fact, OHIP demonstrated more than a threefold increase in sensitivity (21%–74%). Conversely, CORR had substantially lower sensitivity (77%–44%), with a very slight increase in PPV from 92% to 98% (Table S2). Further subgroup analysis conducted for only kidney transplants showed a similar trend, with increased PPV and sensitivity for CIHI‐DAD‐p and OHIP, and decreased sensitivity for CORR. OHIP's sensitivity again improved almost threefold, from 30% to 87%. CORR's decline in sensitivity was not as remarkable compared to its decline for all solid organ transplants, decreasing from 88% to 78% (Table S3). Overall, when comparing the sensitivity and PPV of cohorts that entered the study before and after the switch to ICD‐10 and CCI codes, our data demonstrated an improvement in sensitivity and PPV for CIHI‐DAD‐p and OHIP, and a decline in sensitivity and PPV for CORR. This analysis also showed a decrease in sensitivity of CORR, most notably when all solid organ transplant types were analyzed together.

## 4 Discussion

In this population‐based cohort study, we assessed the accuracy of healthcare administrative databases in identifying pediatric patients with solid organ transplants, including kidney, heart, liver and lung transplants. The most promising single database algorithm tested showed that CIHI‐DAD procedural codes yield a sensitivity of 91% and PPV of 93% for all pediatric solid organ transplants, compared with the reference standard. CIHI‐DAD‐p outperformed CORR and OHIP, and either did better or the same as other combination database algorithms. CIHI‐DAD‐p was crucial to the performance of combination algorithms, with combinations of CORR and OHIP alone resulting in poor sensitivity and PPV.

The observed differences in sensitivity between the transplant center and the databases in our study can be attributed to variations in data collection methods. CIHI‐DAD employs nationally standardized protocols for chart abstraction, with all abstractors undergoing comprehensive standardized training for conformity to strict guidelines [16]. This methodological rigor likely explains the high sensitivity and PPV of CIHI‐DAD‐p. While the same might have been expected for CIHI‐DAD diagnostic codes as well, we found that these were unsuitable for analysis, as < 6 individuals with a CIHI‐DAD diagnostic code had a transplant date aligned with the SickKids database. This discrepancy may stem from the fact that a transplant may be coded in the patient's chart after the transplant surgery for a subsequent admission and does not necessarily reflect the date of the transplant. In contrast to CIHI‐DAD, the OHIP database relies on physician billing claims, which may not always be incentivized if a physician works in a center that is salaried, thereby leading to potential underreporting. Lastly, the CORR database relies on voluntary transplant center submissions, leading to variability in data completeness and sensitivity depending on the extent of information provided by each center [20].

The observed difference in PPV between the transplant center data and the administrative databases suggests the identification of false positives. This discrepancy may indicate incorrect labeling by the administrative databases, given their reliance on physician claims and data abstraction. Other explanations to consider include the following: (1) multiorgan transplant recipients who were coded as having received only a single organ transplant, (2) patients whose transplant procedures were aborted intraoperatively but were nonetheless recorded as completed transplants, and (3) individuals who underwent their transplant at another pediatric center in Canada. Importantly, data go to various sources and each time, there may be variability in coding and algorithms which are typically developed for adult end‐stage disease and may not account for some pediatric cases.

Interestingly, unlike our findings, one study of adult kidney transplant recipients found CIHI‐DAD‐p, CORR and OHIP to be equally reliable [21]. Differences in billing structures between adult physicians and pediatricians, could explain this discrepancy. In addition, the adult study covered a shorter period (2008–2011) and analyzed fewer codes per dataset. No pediatric studies were available for comparison.

This study is subject to limitations. Data from the reference standard ranges from 1991 to 2011 and we cannot attest to the validity of reported databases beyond the study period. However, our additional analysis comparing CIHI‐DAD procedural code sensitivity and PPV pre and post CCI implementation showed minimal change, which might imply that CIHI‐DAD procedural codes are not impacted by changes made after the CCI implementation. Second, these algorithms are tested against a reference standard which includes data from only one pediatric transplant center in Ontario, and the algorithms may perform differently in other regions with differing practice patterns. Third, this study validates Canadian health administrative data algorithms and results may not be applicable in jurisdictions that use other data sources. Lastly, there is potential for changing validity overtime as adjustments are made to the codes in these databases, as well as to how information is collected. Such bias can be actively prevented by continually updating validation data as changes are made to the databases.

In summary, this is the first study to validate the use of health administrative data to identify pediatric solid organ transplants (kidney, heart, liver, and lung) in Canada. Data on short‐ and long‐term outcomes of children living with solid organ transplants have been challenged by small cohort sizes, loss to follow‐up, insufficient long‐term data, and reliance on single‐center studies [1, 2, 3, 4, 5]. We demonstrate that CIHI‐DAD procedural codes show high sensitivity and PPV for all solid organ transplants, proving highly reliable compared to transplant center records. Our findings suggest that a national administrative database using procedural codes can reliably identify pediatric solid organ transplant recipients, enabling further research to improve clinical care, patient outcomes, and healthcare policy.

## Funding

Dr. Naylor is supported by a Health System Impact Embedded Early Career Researcher Award Canadian Institutes of Health Research. This study was supported by ICES, which is funded by an annual grant from the Ontario Ministry of Health (MOH) and the Ministry of Long‐Term Care (MLTC) and project grant from the Canadian Institutes of Health Research (Funding Reference Number 178016).

## Conflicts of Interest

The authors declare no conflicts of interest.

## Supporting information


**Data S1:** petr70245‐sup‐0001‐DataS1.docx.

## Data Availability

The dataset from this study is held securely in coded form at ICES. While legal data sharing agreements between ICES and data providers (e.g., healthcare organizations and government) prohibit ICES from making the dataset publicly available, access may be granted to those who meet prespecified criteria for confidential access, available at www.ices.on.ca/DAS (email: das@ices.on.ca). The full dataset creation plan and underlying analytic code are available from the authors upon request, understanding that the computer programs may rely upon coding templates or macros that are unique to ICES and are therefore either inaccessible or may require modification.
