# Supplementary material for: Validating Recipients of Pediatric Solid Organ Transplant Using Administrative Healthcare Data
Source: Pediatr Transplant. 2025 Dec 8;29(8):e70245. doi: 10.1111/petr.70245 (PMC12686849; doi:10.1111/petr.70245)
Supplement: Supplementary file 1 — Data S1: petr70245‐sup‐0001‐DataS1.docx. [file PETR-29-e70245-s001.docx]

**Supplementary Content**

**Supplementary Figure S1**: Flowchart of study cohort creation after screening for exclusion criteria

**Supplementary Figure S2:** Flowchart of database combinations

**Supplementary Table S1:** Pediatric solid organ transplant recipients identified by administrative databases

**Supplementary Table S2:** Sensitivity and PPV of individual databases in identifying all solid organ transplant recipients when patients entered the cohort prior to April 1, 2002 vs. on or after April 1, 2002.

**Supplementary Table S3:** Sensitivity and PPV of individual databases in identifying kidney transplant recipients when patients entered the cohort prior to April 1, 2002 vs. on or after April 1, 2002.

**Appendix S1**. The RECORD Statement—Checklist of Items, Extended From the STROBE Statement, That Should Be Reported in Observational Studies Using Routinely Collected Health Data

**Appendix S2**. Administrative Database Codes to Identify Pediatric Solid Organ Transplant Recipients

**Supplementary Figure S1.** Flowchart of study cohort creation after screening for exclusion criteria.

**Initial Cohorts**

1. Reference Standard (n = 839)

2. CORR (n = 11,624)

3. CIHI-DAD procedural (n = 12,729)

4. CIHI-DAD diagnostic (n= 14,409)

5. OHIP (n = 13,252)

**Excluded due to invalid IKN, missing DOB, missing sex, death before index date, non-Ontario resident**

1. Reference Standard (n = 7)

2. CORR (n = 61)

3. CIHI-DAD procedural (n = 68)

4. CIHI-DAD diagnostic(n= 52)

5. OHIP (n = 83)

**Excluded due to age ≥18**

1. Reference Standard (n = 7)

2. CORR (n = 10,945)

3. CIHI-DAD procedural (n = 11,735)

4. CIHI-DAD diagnostic (n= 13,428)

5. OHIP (n = 12,498)

**Excluded due to transplant not performed at SickKids/multi-organ transplant**

1. Reference Standard (n = 0)

2. CORR (n = 104)

3. CIHI-DAD procedural (n = 122)

4. CIHI-DAD diagnostic (n= 275)

5. OHIP (n = 212)

**Final Cohorts**

1. Reference Standard (n = 825)

2. CORR (n = 514)

3. CIHI-DAD procedural (n = 804)

4. CIHI-DAD diagnostic (n = 654)

5. OHIP (n = 459)

Abbreviations: CORR, Canadian Organ Replacement Register; CIHI-DAD, Canadian Institute for Health Information Discharge Abstract Database; DOB, Date of Birth; IKN, ICES Key Number; OHIP, Ontario Health Insurance Plan

**Supplementary Figure S2:** Flowchart of database combinations

| **Primary Databases** | **Database combinations** |
| --- | --- |

1. CIHI-DAD-p OR CORR

2. CIHI-DAD-p AND CORR

Canadian Institute for Health Information Discharge Abstract Database procedural codes (**CIHI-DAD-p**)

7. CIHI-DAD-p OR

CORR OR

OHIP

3. CIHI-DAD-p OR OHIP

4. CIHI-DAD-p AND OHIP

Canadian Organ Replacement Register (**CORR**)

Ontario Health Insurance Plan (**OHIP**)

5. CORR OR OHIP

6. CORR AND OHIP

Abbreviations: CORR, Canadian Organ Replacement Register; CIHI-DAD-p, Canadian Institute for Health Information Discharge Abstract Database procedural codes; OHIP, Ontario Health Insurance Plan

**Supplementary Table S1:** Pediatric solid organ transplant recipients identified by administrative databases

|  |  | **CORR** | |
| --- | --- | --- | --- |
|  |  | 0 | 1 |
| **Centre-specific** | 0 |  | 29 |
|  | 1 | 340 | 485 |
|  |  |  |  |
|  |  | **CIHI-DAD-p** | |
|  |  | 0 | 1 |
| **Centre-specific** | 0 |  | 57 |
|  | 1 | 78 | 747 |
|  |  |  |  |
|  |  | **OHIP** | |
|  |  |  |  |
| **Centre-specific** | 0 |  | 41 |
|  | 1 | 407 | 418 |

Abbreviations: CORR, Canadian Organ Replacement Register; CIHI-DAD-p, Canadian Institute for Health Information Discharge Abstract Database procedural codes; OHIP, Ontario Health Insurance Plan

**Supplementary Table S2:** Sensitivity and PPV of individual databases in identifying all solid organ transplant recipients when patients entered the cohort prior to April 1, 2002 vs. on or after April 1, 2002.

|  | All transplants, sensitivity  (n, 95% CI) | | All transplants, PPV  (n, 95% CI) | |
| --- | --- | --- | --- | --- |
|  | **Pre** | **Post** | **Pre** | **Post** |
| CORR | 0.77  (0.73, 0.81) | 0.44  (0.40, 0.49) | 0.92  (0.89, 0.95) | 0.98  (0.96, 1.00) |
| CIHI-DAD-p | 0.87  (0.83, 0.90) | 0.94  (0.91, 0.96) | 0.89  (0.86, 0.92) | 0.96  (0.94, 0.98) |
| OHIP | 0.21  (0.17, 0.26) | 0.74  (0.70, 0.78) | 0.85  (0.77, 0.92) | 0.93  (0.90, 0.95) |

Abbreviations: CI, Confidence Interval; CORR, Canadian Organ Replacement Register; CIHI-DAD-p, Canadian Institute for Health Information Discharge Abstract Database procedural codes; OHIP, Ontario Health Insurance Plan; PPV, Positive Predictive Value

**Supplementary Table S3:** Sensitivity and PPV of individual databases in identifying kidney transplant recipients when patients entered the cohort prior to April 1, 2002 vs. on or after April 1, 2002.

|  | Kidney transplant, sensitivity (n, 95% CI) | | Kidney transplant, PPV  (n, 95% CI) | |
| --- | --- | --- | --- | --- |
|  | **Pre** | **Post** | **Pre** | **Post** |
| CORR | 0.88  (0.82, 0.93) | 0.78  (0.71, 0.84) | 0.90  (0.85, 0.94) | 0.99  (0.97, 1.00) |
| CIHI-DAD-p | 0.89  (0.85, 0.94) | 0.97  (0.94, 0.99) | 0.88  (0.83, 0.93) | 0.99  (0.97, 1.00) |
| OHIP | 0.30  (0.23, 0.36) | 0.87  (0.82, 0.92) | 0.81  (0.71, 0.90) | 0.94  (0.91, 0.98) |

Abbreviations: CI, Confidence Interval; CORR, Canadian Organ Replacement Register; CIHI-DAD-p, Canadian Institute for Health Information Discharge Abstract Database procedural codes; OHIP, Ontario Health Insurance Plan; PPV, Positive Predictive Value

**Appendix S1**. The RECORD Statement—Checklist of Items, Extended From the STROBE Statement, That Should Be Reported in Observational Studies Using Routinely Collected Health Data

|  | **Item No.** | **STROBE items** | **Location in manuscript where items are reported** | **RECORD items** | **Location in manuscript where items are reported** |
| --- | --- | --- | --- | --- | --- |
| **Title and abstract** | | | | | |
|  | 1 | (a) Indicate the study’s design with a commonly used term in the title or the abstract (b) Provide in the abstract an informative and balanced summary of what was done and what was found | Abstract, page 2  Abstract, page 2 | RECORD 1.1: The type of data used should be specified in the title or abstract. When possible, the name of the databases used should be included.  RECORD 1.2: If applicable, the geographic region and timeframe within which the study took place should be reported in the title or abstract.  RECORD 1.3: If linkage between databases was conducted for the study, this should be clearly stated in the title or abstract. | Abstract, page 2  Abstract, page 2  Abstract, page 2 |
| **Introduction** | | | | | |
| Background rationale | 2 | Explain the scientific background and rationale for the investigation being reported | Page 3-4, Introduction |  |  |
| Objectives | 3 | State specific objectives, including any prespecified hypotheses | Page 3-4, Introduction |  |  |
| **Methods** | | | | | |
| Study Design | 4 | Present key elements of study design early in the paper | Page 4-5, Methods |  |  |
| Setting | 5 | Describe the setting, locations, and relevant dates, including periods of recruitment, exposure, follow-up, and data collection | Page 4-6, Methods |  |  |
| Participants | 6 | *(a) Cohort study* - Give the eligibility criteria, and the sources and methods of selection of participants. Describe methods of follow-up  *Case-control study* - Give the eligibility criteria, and the sources and methods of case ascertainment and control selection. Give the rationale for the choice of cases and controls  *Cross-sectional study* - Give the eligibility criteria, and the sources and methods of selection of participants  *(b) Cohort study* - For matched studies, give matching criteria and number of exposed and unexposed  *Case-control study* - For matched studies, give matching criteria and the number of controls per case | Page 4-6, Methods | RECORD 6.1: The methods of study population selection (such as codes or algorithms used to identify subjects) should be listed in detail. If this is not possible, an explanation should be provided.  RECORD 6.2: Any validation studies of the codes or algorithms used to select the population should be referenced. If validation was conducted for this study and not published elsewhere, detailed methods and results should be provided.  RECORD 6.3: If the study involved linkage of databases, consider use of a flow diagram or other graphical display to demonstrate the data linkage process, including the number of individuals with linked data at each stage. | Page 5-6, Methods  N/A  Supplementary Figure S1 in Supplementary Data |
| Variables | 7 | Clearly define all outcomes, exposures, predictors, potential confounders, and effect modifiers. Give diagnostic criteria, if applicable. | Appendix 2 in Supplementary Data | RECORD 7.1: A complete list of codes and algorithms used to classify exposures, outcomes, confounders, and effect modifiers should be provided. If these cannot be reported, an explanation should be provided. | Appendix 2 in Supplementary Data |
| Data sources/ measurement | 8 | For each variable of interest, give sources of data and details of methods of assessment (measurement).  Describe comparability of assessment methods if there is more than one group | Appendix 2 in Supplementary Data |  |  |
| Bias | 9 | Describe any efforts to address potential sources of bias | Page 4-7, Methods |  |  |
| Study size | 10 | Explain how the study size was arrived at | Page 6, Methods - Population |  |  |
| Quantitative variables | 11 | Explain how quantitative variables were handled in the analyses. If applicable, describe which groupings were chosen, and why | Page 6-7, Methods – Statistical methods |  |  |
| Statistical methods | 12 | (a) Describe all statistical methods, including those used to control for confounding  (b) Describe any methods used to examine subgroups and interactions  (c) Explain how missing data were addressed  (d) *Cohort study* - If applicable, explain how loss to follow-up was addressed  *Case-control study* - If applicable, explain how matching of cases and controls was addressed  *Cross-sectional study* - If applicable, describe analytical methods taking account of sampling strategy  (e) Describe any sensitivity analyses | Page 6-7, Methods – Statistical methods |  |  |
| Data access and cleaning methods |  | .. |  | RECORD 12.1: Authors should describe the extent to which the investigators had access to the database population used to create the study population.  RECORD 12.2: Authors should provide information on the data cleaning methods used in the study. | Page 6, Methods - population  Page 6, Methods – Population, Statistical methods |
| Linkage |  | .. |  | RECORD 12.3: State whether the study included person-level, institutional-level, or other data linkage across two or more databases. The methods of linkage and methods of linkage quality evaluation should be provided. | Page 6, Methods – Statistical methods – Primary analysis |
| **Results** | | | | | |
| Participants | 13 | (a) Report the numbers of individuals at each stage of the study (*e.g.*, numbers potentially eligible, examined for eligibility, confirmed eligible, included in the study, completing follow-up, and analysed)  (b) Give reasons for non-participation at each stage.  (c) Consider use of a flow diagram | Supplementary Figure S1 in Supplementary Data | RECORD 13.1: Describe in detail the selection of the persons included in the study (*i.e.,* study population selection) including filtering based on data quality, data availability and linkage. The selection of included persons can be described in the text and/or by means of the study flow diagram. | Supplementary Figure S1 in Supplementary Data |
| Descriptive data | 14 | (a) Give characteristics of study participants (*e.g.*, demographic, clinical, social) and information on exposures and potential confounders  (b) Indicate the number of participants with missing data for each variable of interest  (c) *Cohort study* - summarise follow-up time (*e.g.*, average and total amount) | Supplementary Figure S1 in Supplementary Data  Supplementary Figure S1 in Supplementary Data  N/A |  |  |
| Outcome data | 15 | *Cohort study* - Report numbers of outcome events or summary measures over time  *Case-control study* - Report numbers in each exposure category, or summary measures of exposure  *Cross-sectional study* - Report numbers of outcome events or summary measures | Page 8, Results |  |  |
| Main results | 16 | (a) Give unadjusted estimates and, if applicable, confounder-adjusted estimates and their precision (e.g., 95% confidence interval). Make clear which confounders were adjusted for and why they were included  (b) Report category boundaries when continuous variables were categorized  (c) If relevant, consider translating estimates of relative risk into absolute risk for a meaningful time period | Page 8-10, Results  N/A  N/A |  |  |
| Other analyses | 17 | Report other analyses done—e.g., analyses of subgroups and interactions, and sensitivity analyses | Page 11, Additional analysis |  |  |
| **Discussion** | | | | | |
| Key results | 18 | Summarise key results with reference to study objectives | Page 11-12, Discussion |  |  |
| Limitations | 19 | Discuss limitations of the study, taking into account sources of potential bias or imprecision. Discuss both direction and magnitude of any potential bias | Page 13, Discussion | RECORD 19.1: Discuss the implications of using data that were not created or collected to answer the specific research question(s). Include discussion of misclassification bias, unmeasured confounding, missing data, and changing eligibility over time, as they pertain to the study being reported. | Page 13, Discussion |
| Interpretation | 20 | Give a cautious overall interpretation of results considering objectives, limitations, multiplicity of analyses, results from similar studies, and other relevant evidence | Page 14, Discussion |  |  |
| Generalisability | 21 | Discuss the generalisability (external validity) of the study results | Page 14, Discussion |  |  |
| **Other Information** | | | | | |
| Funding | 22 | Give the source of funding and the role of the funders for the present study and, if applicable, for the original study on which the present article is based | Page 14-15, Acknowledgements |  |  |
| Accessibility of protocol, raw data, and programming code |  | .. |  | RECORD 22.1: Authors should provide information on how to access any supplemental information such as the study protocol, raw data, or programming code. | Page 15, Data availability statement and supporting information statement |

*Reference: Benchimol EI, Smeeth L, Guttmann A, Harron K, Moher D, Petersen I, Sørensen HT, von Elm E, Langan SM, the RECORD Working Committee. The REporting of studies Conducted using Observational Routinely-collected health Data (RECORD) Statement. *PLoS Medicine* 2015; in press.

*Checklist is protected under Creative Commons Attribution ([CC BY](http://creativecommons.org/licenses/by/4.0/)) license.

**Appendix S2**. Administrative Database Codes to Identify Pediatric Solid Organ Transplant Recipients

| Database | Code | Description |
| --- | --- | --- |
| CIHI-DAD (Diagnostic) | ICD-9: V42.0  ICD-9: V42.7  ICD-9: V42.1  ICD-9: V42.6  ICD-10: Z94.0  ICD-10: Z94.4  ICD-10: Z94.1  ICD-10: Z94.2 | Kidney transplant status  Liver transplant status  Heart transplant status  Lung transplant status  Kidney transplant status  Liver transplant status  Heart transplant status  Lung transplant status |
| CIHI-DAD (Procedural) | CCP: 675  CCP: 6759  CCP: 624  CCP: 6241  CCP: 6249  CCP: 495  CCP: 455  CCI: 1PC85  CCI: 1OA85LAXXK  CCI: 1OA85WLXXJ  CCI: 1OA85WLXXK  CCI: 1HZ85LAXXK  CCI: 1HZ85LAXXL  CCI: 1GR85LAXXJ  CCI: 1GR85LAXXK  CCI: 1GR85VCXXJ  CCI: 1GR85VCXXK  CCI: 1GT85LAXXJ  CCI: 1GT85LAXXK | Transplant of Kidney  Other kidney transplantation  Liver transplant  Auxiliary liver transplant  Other transplant of liver  Heart transplantation  Lung transplant  Transplant, kidney  Transplant, liver of a deceased donor full size liver  Transplant, liver of a living donor split liver  Transplant, liver of a deceased donor split liver (or reduced or reduced paediatric-size liver)  Transplant, heart NEC using homograft  Transplant, heart NEC using xenograft  Transplant, lobe of lung homograft from living donor of single lobe using open approach  Transplant, lobe of lung homograft from deceased donor of single lobe using open approach  Transplant, lobe of lung homograft from living donor of multiple lobes using open approach  Transplant, lobe of lung homograft from deceased donor of multiple lobes using open approach  Transplant, lung NEC using open approach and homograft from living donor  Transplant, lung NEC using open approach and homograft from deceased donor |
| CORR | TREATMENT_CODE=171  Transplanted organ type code:  10  11  12  18  19  20  21  22  23  29  30  40  41  42  48  49 | Acute care hospital, Transplantation, Total care  Kidneys/Dialysis (includes enbloc transplants)  Kidney – Left  Kidney - Right  Kidney - One (from conversion)  Kidney - Two (from conversion)  Liver  Liver - Left Lobe  Liver - Right Lobe  Liver - Lateral Segment  Liver - Two (from conversion)  Heart  Lungs (Bilateral)  Lung - Left  Lung - Right  Lung - One (from conversion)  Lung - Two (from conversion) |
| OHIP | S435  S434  S294  S295  S266  R870  M155  M156 | Kidney transplant  Kidney re-transplant  Deceased donor, liver transplant  Repeat liver transplant  Living donor orthotopic liver transplantation  Orthotopic cardiac transplantation  Lung transplant (one lung)  Repeat lung transplant (one lung) |

Abbreviations: CCI, Canadian Classification of Health Interventions, is the newest national standard for classifying healthcare procedures. It replaced the Canadian Classification of Diagnostic, Therapeutic and Surgical Procedures (CCP) starting in 2001. ICD-9 was adopted in Canada in 1979, and replaced with ICD-10 beginning April 1, 2001. Abbreviations: CORR, Canadian Organ Replacement Register; CIHI-DAD, Canadian Institute for Health Information Discharge Abstract Database; OHIP, Ontario Health Insurance Plan; NEC, not otherwise specified
